# Levels and function of regulatory T cells in patients with polymorphic light eruption: relation to photohardening

**DOI:** 10.1111/bjd.13930

**Published:** 2015-07-30

**Authors:** N. Schweintzger, A. Gruber‐Wackernagel, E. Reginato, I. Bambach, F. Quehenberger, S.N. Byrne, P. Wolf

**Affiliations:** ^1^ Research Unit for PhotodermatologyDepartment of DermatologyMedical University of GrazAuenbrugger Platz 8A‐8036GrazAustria; ^2^ Center for Medical ResearchMedical University of GrazAuenbrugger Platz 8A‐8036GrazAustria; ^3^ Institute for Medical Informatics, Statistics and DocumentationMedical University of GrazAuenbrugger Platz 8A‐8036GrazAustria; ^4^ Cellular Photoimmunology GroupInfectious Diseases and ImmunologySydney Medical SchoolThe Charles Perkins Centre Hub at The University of SydneyAustralia

## Abstract

**Background:**

We hypothesized that regulatory T cells (Tregs) are involved in the immunological abnormalities seen in patients with polymorphic light eruption (PLE).

**Objectives:**

To investigate the number and suppressive function of peripheral Tregs in patients with PLE compared with healthy controls.

**Methods:**

Blood sampling was done in 30 patients with PLE [seeking or not seeking 311‐nm ultraviolet (UV)B photohardening] as well as 19 healthy controls at two time points: TP1, March to June (before phototherapy); and TP2, May to August (after phototherapy). We compared the number of CD4^+^ CD25^high^ CD127^−^FoxP3^+^ Tregs by flow cytometry and their function by assessing *FoxP3* mRNA levels and effector T cell/Treg suppression assays.

**Results:**

Tregs isolated from healthy controls significantly suppressed the proliferation of effector T cells at TP1 by 68% (*P *=* *0·0156). In contrast, Tregs from patients with PLE entirely lacked the capacity to suppress effector T‐cell proliferation at that time point. The medical photohardening seen in 23 patients with PLE resulted in a significant increase in the median percentage of circulating Tregs [both as a proportion of all lymphocytes; 65 6% increase (*P *=* *0·0049), and as a proportion of CD4^+^ T cells; 32.5% increase (*P *=* *0·0049)]. This was accompanied by an increase in the expression of *FoxP3* mRNA (*P *=* *0·0083) and relative immunosuppressive function of Tregs (*P *=* *0·083) comparing the two time points in representative subsets of patients with healthy controls tested. Seven patients with PLE not receiving 311‐nm UVB also exhibited an increase in the number of Tregs but this was not statistically significant. No significant differences in Treg numbers were observed in healthy subjects between the two time points.

**Conclusions:**

An impaired Treg function is likely to play a role in PLE pathogenesis. A UV‐induced increase in the number of Tregs (either naturally or therapeutically) may be a compensatory mechanism by which the immune system counteracts the susceptibility to PLE.

## Abstract 2

**What's already known about this topic?**

- Patients with polymorphic light eruption (PLE) display immunological abnormalities.
- Previous studies have shown that they are resistant to the immune suppressive effects of sunlight.

**What does this study add?**

- We found that the number and suppressive function of regulatory T cells (Tregs) are crucial in the pathogenesis of PLE.
- An increase in Treg levels (after photohardening) might be a compensatory mechanism by which the immune system intends to counteract the susceptibility to PLE formation.

Polymorphic light eruption (PLE) is a common photosensitivity disorder with a known female preponderance.^1^, ^2^, ^3^, ^4^ The disease is characterized by itchy, self‐limiting, nonscarring skin lesions appearing on exposed body sites several hours to days after the first sun exposure of the season. PLE usually appears in early or late spring, but a so‐called ‘hardening effect’ arises as summer progresses, resulting in reduced and less intense lesions.^5^, ^6^ Medical photo(chemo)therapy [including psoralen plus ultraviolet (UV) A, UVB broadband or narrowband 311‐nm UVB] simulates this naturally occurring phenomenon of hardening and aims to induce photoadaption with regulated doses of UV radiation (UVR) without inducing the manifestation of the disease.^7^, ^8^

The immunological events that arise in the skin after UVR lead to activation the of CD4^+^CD25^+^CTLA‐4^+^FoxP3^+^ regulatory T cells (Tregs), controlling inflammation and adaptive immunity by their immunosuppressive capacity.^9^, ^10^, ^11^, ^12^, ^13^ This may prevent autoreactive, inflammatory responses that could develop against modified lipids, proteins and/or DNA following exposure to UVR.^1^, ^2^, ^14^ There is now convincing evidence that Tregs play a major protective role in the pathogenesis of a number of inflammatory skin diseases including psoriasis, atopic dermatitis and lupus.^15^, ^16^, ^17^ The normal immune suppressive response to UVR is defective in patients with PLE,^18^, ^19^, ^20^ which is thought to lead to an inflammatory reaction that results in a recurrent, pruritic skin rash typical of the condition following exposure to sunlight. We hypothesized that patients with PLE might have abnormal Treg levels and/or function.^2^ In this study we show that successful medical photohardening of patients with PLE resulted in a significant increase in the percentage of FoxP3^+^ Tregs in the blood, which was also reflected at the mRNA level. Compared with healthy controls, the suppressive function of Tregs from patients with PLE was defective. However, after phototherapy these patients displayed a trend towards an increased suppressive function of Tregs.

## Patients and methods

### Study setup

This study was conducted at the Photodermatology Unit, Medical University of Graz, Austria to investigate the levels and function of Tregs in patients with PLE and healthy controls (ClinicalTrials.Gov registration number NCT00555178). The following null and alternative hypotheses were tested: *H*_0_, < 30% increase and *H*_A_, ≥ 30% increase in Treg levels comparing baseline with after‐phototherapy treatment. Sample‐size calculations were based on the data from a previous study^21^ and were performed using the Power/Sample Size Calculator (Institute of Medical Statistics, Medical University Vienna, Austria) with an α error of 0·05 and a power of 0·8. By assuming a dropout rate of 10% this resulted in a group size of 23.

The inclusion criteria for the patients with PLE was age above 18 years and good general health status. The diagnosis of PLE had to be confirmed by the patient's history, histological findings and/or phototesting procedures. Physician‐guided patient history leading to diagnosis of PLE included questioning of patients on (i) the typical formation of itchy skin lesions located on sun‐exposed body sites such as the V of the neck, the back of the hands, the outside surface of the arms and lower legs, appearing within hours after sun exposure; (ii) resolution of the lesions without scarring within a few days; (iii) and the weakening or entire disappearance of symptoms as spring and summer progress.

Exclusion criteria included the presence or history of malignant skin tumours, dysplastic naevus syndrome, autoimmune diseases, systemic treatment with steroids or other immunosuppressive drugs (ongoing, within the last 6 months or planned during the study period), antinuclear antibodies (such as ds‐DNA, Ro, La), specific immunotherapy (i.e. hyposensitization treatment; ongoing, within the last 6 months or planned), and pregnancy or breast‐feeding. Patients seeking or not seeking photohardening therapy in spring were recruited. In addition, age‐matched healthy control subjects were enrolled during the same period of time. In the initial study protocol, patients with phototherapy‐responsive diseases (such as psoriasis and atopic dermatitis) represented another study group. Two additional time points in late summer and fall were also part of the protocol for all patient groups. Due to substantial losses in patient follow‐up numbers at those later time points, together with large variability in the group of patients with phototherapy responsiveness, these data have been omitted from the present analysis.

The study was approved by the local Ethical Committee of the Medical University of Graz (No. 18‐116 ex 06/07). All patients and controls gave informed consent and the study was conducted according to the Declaration of Helsinki principles.

### Study subjects and phototherapy characteristics

Thirty patients with PLE were enrolled in this study between 2008 and 2014. Standard 311‐nm UVB photohardening therapy^22^, ^23^ was started in spring (immediately after the first blood collection; TP1, see below) in 23 of the patients (22 females and one male; mean age 37·4 years, range 18–75) two to three times per week for 4–9 weeks (median, 6 weeks). Seven patients with PLE (all female; mean age 42·0 years, range 21–56) chose not to receive photohardening therapy and represented an additional study group (see Supplementary Table S1 for details about patients and phototherapy). Nineteen healthy subjects (15 females and four males; mean age 38·6 years, range 24–61) without a history of PLE were enrolled and served as controls. One patient with PLE not seeking phototherapy and one healthy volunteer dropped out of the study after first blood drawing, each of them due to personal reasons. Their data are not included in the patient demographics and study analysis (Supplementary Fig. S1).

### Blood sample collection and processing

Blood was collected using lithium–heparin tubes (Vacuette^®^; Greiner Bio‐One, Kremsmünster, Austria) before the start of photohardening in March to June and within 48 h of the (pen)ultimate exposure of the photohardening treatment from May to August. The first time point (pre‐photohardening) is labelled TP1 and the second time point (post‐photohardening) is labelled TP2 in this report and its figures. The average time that elapsed between the first and second blood withdrawal was 48·0 days (photohardened PLE group), 57·7 days (nonphotohardened PLE group) and 55·4 days (controls). Blood materials were available from all patients for flow cytometry analysis and from a subset of eight patients and eight healthy controls for suppression assays and mRNA analysis.

### Peripheral blood mononuclear cells isolation and flow cytometry

Peripheral blood mononuclear cells (PBMCs) were isolated by density gradient centrifugation using Lymphoprep^TM^ (Axis‐Shield, Heidelberg, Germany) for processing and analysis by flow cytometry, reverse transcriptase–polymerase chain reaction (RT‐PCR) and *in vitro* Treg suppression assays. The following antibodies were used for sorting of PBMCs using a FACSAria IIu cell sorter (BD Biosciences, San Jose, CA, U.S.A.): fluorescein–isothiocyanate (FITC)‐conjugated antihuman CD4 (clone RPA‐T4), PE‐Cy7‐conjugated antihuman CD25 (M‐A251), phycoerythrin (PE)‐conjugated antihuman CD127 (clone hIL‐7R‐M21), all from eBioscience (Vienna, Austria). We used PE‐conjugated antihuman CD127 (clone hIL‐7R‐M21), FITC‐conjugated antihuman CD25 (clone 2A3), peridinin chlorophyll protein complex (PerCP)‐conjugated antihuman CD4 (clone SK3) (BD Pharmingen, San Diego, CA, U.S.A.) and allophycocyanin (APC)‐conjugated antihuman FoxP3 (clone PCH101) from eBioscience for quantification of human PBMCs. Data was acquired on a FACSCalibur flow cytometer and analysed with FLOWJO software (version 7.6.5; TreeStar Inc., Ashland, OR, U.S.A.). All plots were pregated on CD4^+^ lymphocytes. The increase in percentage of Tregs as a proportion of either the CD4 subpopulation or the entire lymphocyte subpopulation was calculated by dividing TP2 by TP1.

### Regulatory T‐cell suppression assay

Treg suppression assays with blood samples from the subset of eight patients and eight control subjects were performed as described.^24^ In brief, PBMCs were stimulated with 5 μg mL^−1^ purified low endotoxin/sodium azide‐free mouse antihuman CD3 (clone UCHT1; BD Pharmingen) and 2·5 μg mL^−1^ antihuman CD28 (Clone 28.2) (BioLegend, London, U.K.) for 96 h. Cellular proliferation was measured with a Wallac 1450 MicroBeta^®^ TriLux (Perkin Elmer, Brunn am Gebirge, Austria) via tritiated thymidine incorporation (1 μCi per [^3^H]thymidine; Amersham Biosciences, Piscataway, NJ, U.S.A.) added for the final 16 h of the 96 h incubation. For analysis, the 1 : 1 ratio of effector T cells and Tregs was normalized to the proliferative rate of stimulated effector T cells alone (set to 100%).

### RNA isolation and quantitative reverse transcriptase–polymerase chain reaction

RNA was isolated from PBMCs of a subset of patients with PLE receiving phototherapy and healthy controls (*n *=* *8 each) using the Qiagen fibrous mini kit (Qiagen, Hilden, Germany) and transcribed using First‐Strand cDNA Synthesis kit (Roche, Basel, Switzerland). Quantitative RT‐PCR was performed with primers specific for human *FoxP3* (forward: GCTCTGCACCTTCCCAAAT; reverse: TCTCTGGAGGAGACATTGTGC) and the *GAPDH* gene region as a control (forward and reverse primer, PPH00150E‐200; Qiagen). The reactions were run on a 7900HT Fast Real‐Time PCR System (Life Technologies, Vienna, Austria) using RT^2^ SYBR Green‐qPCR Master Mix (Qiagen). To normalize transcripts to *GAPDH* and to calibrate the fold change the ΔΔ*C*_t_ method was used. Data are presented as fold change comparing TP2 with TP1.

### Statistical analysis

Statistical differences were determined by using the Kruskal–Wallis test, the Wilcoxon signed rank test or the Mann–Whitney *U*‐test, as appropriate for the data. Data presented are expressed as medians with range, medians with 95% confidence intervals (CIs) or box‐and‐whisker plots (Tukey method). All analyses were performed with Prism 5.0 (GraphPad Software, Inc., SD, La Jolla, CA, U.S.A.) or R 3.1.2 (www.r-project.org). Statistical significance was set at *P *<* *0·05.

## Results

### Regulatory T‐cell numbers are increased in patients with polymorphic light eruption after photohardening treatment

**Figure 1:**
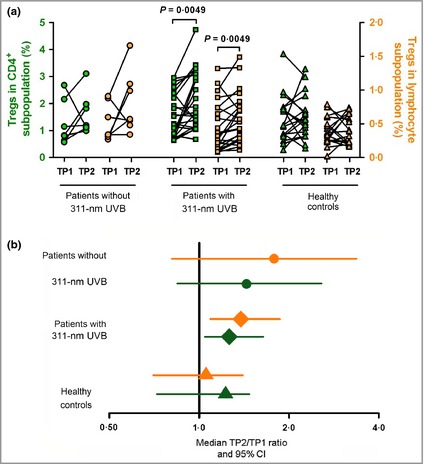
Photohardening treatment increases the number of Tregs in patients with PLE. Peripheral blood mononuclear cells of patients and healthy controls were stained with antibodies for CD4, CD127, CD25 and FoxP3. (a) Percentages of Tregs as a proportion of CD4^+^ cells (green symbols) or all lymphocytes (orange symbols) in PLE patients with or without 311‐nm UVB compared with healthy individuals at TP1 and TP2. (b) Median TP2 : TP1 ratio (± 95% CI) for the percentage of Tregs in CD4^+^ (green symbols) and lymphocyte (orange symbols) subpopulations from each of the three subject groups. Datasets were logarithmized before calculation of ratios. Patients without 311‐nm UVB,* n *=* *7; patients with 311‐nm UVB,* n *=* *23; healthy controls, *n *=* *19. *P*‐values were determined by Wilcoxon test. CI, confidence interval; PLE, polymorphic light eruption; TP1, time point 1 (before phototherapy); TP2, time point 2 (after phototherapy); Tregs, regulatory T cells; UVB, ultraviolet B.

To study the role of Tregs in PLE, we first investigated their numbers by staining PBMCs of patients and controls for CD4, CD25, CD127 and FoxP3 before (Supplementary Fig. S2a) and after (Supplementary Fig. S2b) photohardening treatment. Flow cytometry analysis of PBMC from patients with PLE before (TP1) and after (TP2) medical photohardening revealed a significant increase in the median percentage of CD4^+^CD25^+^FoxP3^+^ Tregs in both the CD4^+^ subpopulation [1·26% vs. 1·67% (an increase of 32·5%); *P *=* *0·0049] and the total lymphocyte population [0·32% vs. 0·53% (an increase of 65·6%); *P *=* *0·0049] (see Fig. 1a and for descriptive statistics, see Supplementary Table S2). There was no statistically significant change in the number of Tregs in the other two groups when comparing TP1 with TP2 (Fig. 1a and Supplementary Table S2). Changes in Treg numbers for the three different groups are depicted in Figure 1b. These were calculated by plotting the median ratio and 95% CI between TP2 and TP1 for patients with PLE with phototherapy, patients with PLE without phototherapy and healthy control subjects. Any number above 1 is representative of an increase in Tregs during the time course. The only ratio to show a significant increase was that for patients with PLE undergoing photohardening therapy with 1·26 (95% CI, 1·04–1·64; *P *=* *0·0083) as a proportion of CD4^+^ T cells, and 1·38 (95% CI, 1·09–1·86; *P *=* *0·0015) as a proportion of all lymphocytes. However, a Kruskal–Wallis test revealed no significant differences in Treg levels among the three groups at baseline (TP1) and at TP2. There were no significant differences in total leucocyte or lymphocyte numbers comparing patients with PLE with healthy control subjects at either TP1 or TP2 or between the two time points (data not shown).

### Regulatory T‐cell function is significantly impaired during spring in patients with polymorphic light eruption

**Figure 2:**
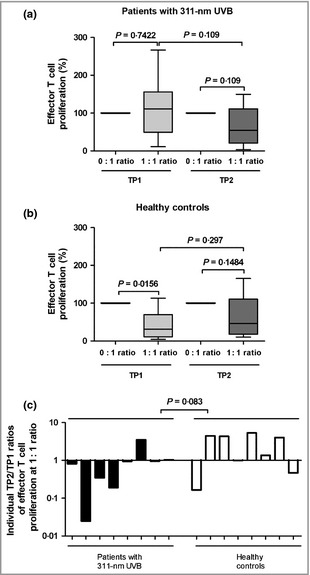
Tregs from patients with PLE have a reduced immune suppressive capacity. Treg suppression assays from (a) patients with PLE and (b) healthy controls at TP1 and TP2. Data shown are box‐and‐whisker plots (Tukey method) of proliferation rates, normalized to effector T‐cell proliferation alone (0 : 1 ratio = 100%) for patients with PLE and healthy controls. The 1 : 1 ratio represents the results of co‐culture of the same number of effector T cells (CD4^+^ CD25^−^ CD127^+^) and Tregs (CD4^+^ CD25^+^ CD127^−^). (c) Individual TP2 : TP1 median ratios of T effector cell proliferation., *n *=* *8. *P*‐values were determined by Wilcoxon test (a,b) and Mann–Whitney *U*‐test (c). PLE, polymorphic light eruption; TP1, time point 1 (before phototherapy); TP2, time point 2 (after phototherapy); Tregs, regulatory T cells.

We also investigated the Treg function in a subset of eight patients receiving medical photohardening and eight healthy control subjects (see Supplementary Fig. S1) by measuring their capacity to inhibit T‐cell proliferation. This subset of patients and control subjects was representative for the entire patient and control group as similar courses of Treg levels were observed for the subsets as shown for the entire groups in Figure 1a by comparing pre‐ vs. post‐phototherapy determined by flow cytometry (i.e. a similar increase in the subset of patients with PLE and no significant change in the subset of control subjects; data not shown). Tregs from healthy controls significantly suppressed the median proliferation of CD3/CD28‐stimulated effector T cells at TP1 by 68% (*P *=* *0·0156) (100% proliferation at 0 : 1 ratio of effector T cells compared with 32% proliferation at a 1 : 1 ratio; Fig. 2b). In contrast, Tregs from patients with PLE lacked any capacity to suppress effector T‐cell proliferation at this time point (Fig. 2a). No significant suppression of effector T‐cell proliferation was observed at TP2 in any group of subjects. However, when the median suppressive activity over the time course was analysed we observed a change in Treg‐mediated suppression in patients with PLE from 111% at TP1 to 54% at TP2. In contrast to healthy controls, where the suppressive activity over the same time course changed from 32% to 46%. For comparison, we expressed this as a ratio of the median Treg suppression at TP2 with that at TP1 (Fig. 2c). Ratios below 1 indicate a rise in suppressive activity whereas ratios above 1 indicate a fall. Analysis of individual ratios confirmed a trend towards a difference between patients receiving 311‐nm UVB and healthy controls displaying a TP2/TP1 ratio with a median of 0·87 and 2·67, respectively (*P *=* *0·083; Fig. 2c).

### *FoxP3* mRNA levels are significantly upregulated in patients with polymorphic light eruption after phototherapy

**Figure 3:**
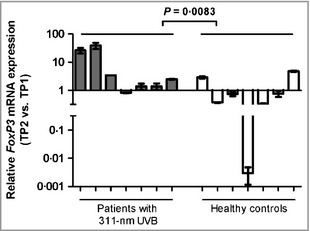
311‐nm UVB upregulates *FoxP3* mRNA in patients with PLE. RT‐PCR results from peripheral blood mononuclear cells of patients and healthy controls are shown as fold change of mRNA (TP2–TP1) (median with range). The ΔΔ*C*_t_ method was used to normalize transcripts to *GAPDH*. Patients with 311‐nm UVB,* n *=* *7; healthy controls, *n *=* *7. *P*‐values were determined by Mann–Whitney *U*‐test. PLE, polymorphic light eruption; RT‐PCR, reverse transcriptase–polymerase chain reaction; UVB, ultraviolet B.

Because higher expression levels of FoxP3 correlate with enhanced Treg suppressive capacity,^25^ we investigated whether medical photohardening therapy affects *FoxP3* mRNA levels in patients with PLE by quantitative RT‐PCR. Transcripts were normalized to *GAPDH* and compared with TP1 for patients with PLE and controls (the same subset of patient and control groups were used as shown in Figure 2c; one patient and one healthy control subject had RNA yields that were too low to be included in this analysis). Relative *FoxP3* mRNA levels were upregulated after photohardening in six of seven patients with PLE (Fig. 3). In contrast, the amount of *FoxP3* mRNA decreased in five of seven healthy individuals between the two time points. This was a statistically significant difference between the two groups (*P *=* *0·0083).

## Discussion

Photohardening therapy given to patients with PLE in spring enables them to tolerate their first high dose of sunlight later in the season with minimal or no eruption. We herewith provide evidence that despite having the same numbers of circulating Tregs as control subjects at the start of spring, Tregs of patients with PLE have an impaired suppressive function (Fig. 2a). This implicates Tregs in the pathogenesis of PLE and potentially explains why these patients are resistant to the normal immune suppressive properties of UVR. Because activation of Tregs is a major way in which UVR suppresses cutaneous immunity, we hypothesized that successful UVR‐induced therapeutic hardening was efficacious, in part, by boosting Treg numbers and/or function. Supporting this hypothesis, we have discovered that medical photohardening with 311‐nm UVB not only significantly increased the numbers of Tregs (Fig. 1a and Supplementary Table S2), but also increased Treg expression of *FoxP3* mRNA, an indicator of increased suppressive function (Fig. 3). A similar increase in the level of Tregs, although not statistically significant, was also observed in patients not receiving medical phototherapy (Fig. 1). This most likely has occurred through hardening by exposure to natural sunlight. Alternatively, a seasonal effect independent of UVR exposure may have caused these changes in both groups of patients compared with healthy control subjects. A limitation of our study is the unequal group size of patients with PLE and further, that the number of enrolled patients without phototherapy was rather low.

The precise reason why patients with PLE have normal numbers of Tregs with suboptimal suppressor capabilities is unknown and the UVR‐induced cutaneous signal that normalizes UVR‐immune suppression in PLE remains to be determined. One possibility is that genetic factors might play a role.^26^, ^27^, ^28^, ^29^, ^30^ Genetic regulation might alter the normal response to UVR in patients through modulation of the immune suppressive function of patients directly by affecting Tregs or indirectly by affecting cells or mediators that recruit or activate Tregs.

For instance, glutathione‐S‐transferase (GST), which is an enzyme that detoxifies reactive oxygen species (ROS), was reported to exert a possible protective effect against PLE because the carrier frequency of the GSTP1 allele was found to be lower in patients than in controls, which would support an involvement of ROS in the pathogenesis of the disease.^31^ This was in contrast to another study that could not find an association between the GST gene family and PLE.^32^ Guarrera and Rebora investigated the hydrosoluble antioxidant capacity which included uric acid, bilirubin, vitamin C, thyols and glutathione and found decreased rates in patients with PLE and moreover lower values in female patients and controls compared with males, which increased in diseased women, with age.^33^ That PLE has a disproportionately higher incidence in young women is known, but the reason is unclear. Sex hormones like 17beta‐estradiol prevent UVR‐induced suppression of contact hypersensitivities through limiting interleukin (IL)‐10 secretion from keratinocytes.^34^, ^35^ This is substantiated by a study of Widyarini *et al*.^36^ who demonstrated a natural photoimmunoprotective role of the oestrogen receptor, as blocking it exacerbated the immune suppression from solar‐simulated UVR. Further studies are required to investigate a possible gene link to sex influence which certainly may not be solely responsible for the pathogenesis of PLE, as our control group, which was well matched and mostly composed of women, did exhibit a differential Treg regulation.

Clues from murine models inform us that dermal mast cell density determines one's susceptibility to UVR immune suppression.^37^ This in turn is dependent on the migration of mast cells into and away from the skin to activate suppressor cells in the local draining lymph nodes.^38^ While the precise mechanism by which dermal mast cells mediate UVR‐induced immune suppression is not known, their production of immunoregulatory IL‐10 following UVR is involved.^39^, ^40^ Alternatively, mast cells might activate Tregs that are required to maintain peripheral tolerance.^41^, ^42^ Intriguingly, we recently made the observation that photohardening increased the numbers of mast cells in the papillary dermis of patients with PLE.^43^ This was accompanied by a recovery of neutrophil responsiveness to the chemoattractants leukotriene B4 and formyl‐methionyl‐leucyl‐phenylalanin.^44^ That PLE is linked to low numbers of Tregs is supported by a recent observation by Gambichler *et al*.,^45^ showing a low Treg infiltration together with a decreased expression of the immunoregulatory factors transforming growth factor‐β1, IL‐10 and receptor activator of nuclear factor‐ĸB ligand in UVA1‐induced skin lesions of patients with PLE. Furthermore, Gruber‐Wackernagel *et al*.^46^ have shown that lesional skin from patients with PLE contained relatively low numbers of Foxp3^+^ cells in the dermal infiltrate compared with calcipotriol‐pretreated skin, which showed less clinical PLE severity. The observation that 311‐nm UVB increased Treg levels in patients with PLE is consistent with the results of a recent study in patients with various skin diseases who showed an increase in peripheral Tregs after treatment with 311‐nm UVB.^47^ The mechanism by which UVR photohardening increases the level and function of Tregs may involve the production of vitamin D. Indeed, its supplementation has been shown to be associated with significantly increased numbers of Tregs in apparently healthy individuals.^48^, ^49^ That said, a previous study^22^ from our laboratory has indicated that 311‐nm UVB hardening was capable of increasing serum 25‐hydroxyvitamin‐D3 levels which may be low in patients with photosensitivity and cases of photodermatoses, including PLE.^50^, ^51^, ^52^

We conclude that a decreased function of Tregs might play a role in the formation of PLE and an increase in Treg numbers might be a compensatory mechanism by which the immune system intends to counteract the susceptibility to PLE formation.

## Supporting information


**Fig S1.** Flow diagram showing numbers of patients with polymorphic light eruption and healthy controls at each stage of the study.

**Fig S2.** Peripheral blood mononuclear cells of patients and healthy controls were stained with antibodies for CD4, CD127, CD25 and FoxP3.

**Table S1.** Characteristics of PLE patients.

**Table S2.** Median percentages of CD4^+^CD25^high^CD127^‐^FoxP^3^^+^ Tregs in PLE patients and healthy controls, as assessed by flow cytometry.
