# Supplementary material for: Levels and function of regulatory T cells in patients with polymorphic light eruption: relation to photohardening
Source: Br J Dermatol. 2015 Jul 30;173(2):519–26. doi: 10.1111/bjd.13930 (PMC4564948; doi:10.1111/bjd.13930)
Supplement: Supplementary file 1 — Fig S1. Flow diagram showing numbers of patients with polymorphic light eruption and healthy controls at each stage of the study. [file BJD-173-519-s001.docx]

**
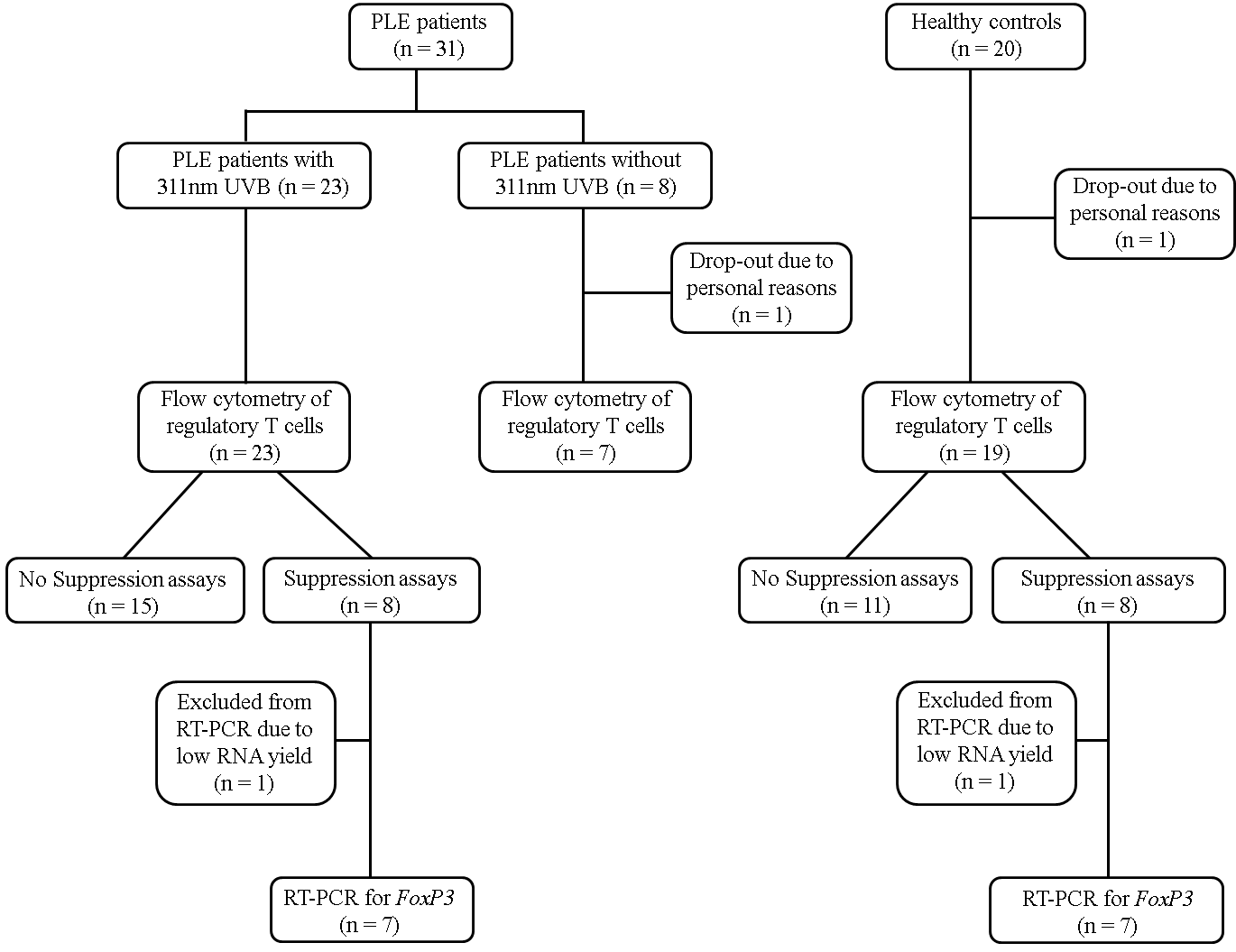
**

**Fig S1.** Flow diagram showing numbers of PLE patients and healthy controls at each stage of the study.
