# Supplementary material for: Levels and function of regulatory T cells in patients with polymorphic light eruption: relation to photohardening
Source: Br J Dermatol. 2015 Jul 30;173(2):519–26. doi: 10.1111/bjd.13930 (PMC4564948; doi:10.1111/bjd.13930)
Supplement: Supplementary file 2 — Fig S2. Peripheral blood mononuclear cells of patients and healthy controls were stained with antibodies for CD4, CD127, CD25 and FoxP3. [file BJD-173-519-s002.docx]

**
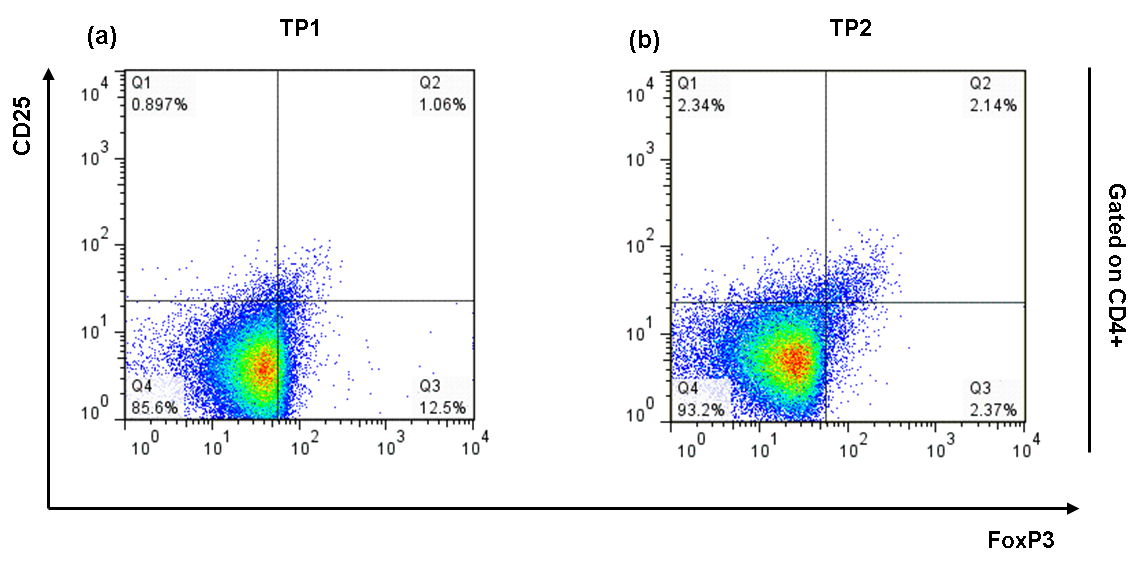
**

**Fig S2.** PBMCs of patients and healthy controls were stained with antibodies for CD4, CD127, CD25 and FoxP3. Cells were pregated on CD4+ for the analysis. Phenotypic analysis of peripheral Tregs from a PLE patient receiving 311nm UVB at (a) TP1 and (b) TP2 by flow cytometry.
