# Supplementary material for: Levels and function of regulatory T cells in patients with polymorphic light eruption: relation to photohardening
Source: Br J Dermatol. 2015 Jul 30;173(2):519–26. doi: 10.1111/bjd.13930 (PMC4564948; doi:10.1111/bjd.13930)
Supplement: Supplementary file 3 — Table S1. Characteristics of PLE patients. [file BJD-173-519-s003.docx]

Table S1. Characteristics of PLE patients.

| **Characteristics** | **Patients without 311nm UVB** | **Patients with 311nm UVB** |
| --- | --- | --- |
| **Gender** | f: 7; m: 0 | f: 22; m: 1 |
| **Age in years (range)** | 42 (21-56) | 37.4 (18-75) |
| **Skin phototype*****, n** | II: 1; III: 6 | II: 5; III: 18 |
| **Mean disease duration in years (range)** | 14 (2-35) | 10 (1-30) |
| **Establishment of diagnosis** | **PGPH: 7; CE: 1; P: 0; H: 0 | PGPH: 23; CE: 7; P: 8; H: 1 |
| **Results of photoprovocation** | nd | ***UVA: 1; UVA/UVB: 4; SSR: 1; neg: 2; nd: 15 |
| **Pathology of PLE lesions** | ****m: 1; mp: 4; pap: 1; pv: 1 | m: 0; mp: 9; pap: 11; pv: 3 |
| **Predilection body site** | V-neck: 7; arm: 3; forearm: 4; thighs: 3; abd.: 2 | V-neck: 21; arm: 12; forearm: 7; thighs: 7 |
| **Mean no. of exposures**  **(range)** | none | 17.6 (11-20) |
| **Mean weeks of treatment (range)** | none | 6.4 (4-9) |
| **Mean starting dose in J/cm^2^ (range)** | none | 0.3 (0.2-0.5) |
| **Mean total dose in J/cm^2^ (range)** | none | - 1. (5.76-20.6) |
| **Phototherapy effectiveness** | na | *****ve: 4; me: 2;  no-FU: 10;  p-FU: 7 (ve to me) |

* According to Fitzpatrick classification; ** PGPH = Physician-guided patient history, as described in Patients and Methods; CE = Clinical examination at presentation of the disease; P = Photoprovocation; H = Histology; *** UVA = UVA positive; UVB = UVB positive; SSR = solar simulated radiation positive; neg = no skin reaction after laboratory photoprovocation with near-erythematogenic daily exposure up to 4 days with UVA or UVB; nd = not done; ****m = macular; mp = maculopapular; pap = papular; pv = papulovesicular; abd. = abdomen; ***** ve = very effective; me = moderately effective; no-FU = no follow-up data; p-FU: previous follow-up data showed the efficiency of earlier photohardening which was very effective in two patients and very to moderately effective in five patients. na, not applicable.
