# Supplementary material for: Levels and function of regulatory T cells in patients with polymorphic light eruption: relation to photohardening
Source: Br J Dermatol. 2015 Jul 30;173(2):519–26. doi: 10.1111/bjd.13930 (PMC4564948; doi:10.1111/bjd.13930)
Supplement: Supplementary file 4 — Table S2. Median percentages of CD4+CD25highCD127‐FoxP3 + Tregs in PLE patients and healthy controls, as assessed by flow cytometry. [file BJD-173-519-s004.docx]

Table S2. Median percentages of CD4^+^CD25^high^CD127^–^FoxP3^+^ Tregs in PLE patients and healthy controls, as assessed by flow cytometry.

| **Type of subpopulation** | **PLE patients** | | | | | | | | **Healthy**  **controls** | | | |
| --- | --- | --- | --- | --- | --- | --- | --- | --- | --- | --- | --- | --- |
|  | **without 311nm UVB** | | | | **with 311nm UVB** | | | |  |  |  |  |
|  | **time points** | | **percent increase** | **p-value** | **time points** | | **percent increase** | **p-value** | **time points** | | **percent increase** | **p-value** |
|  | TP 1 | TP 2 |  |  | TP 1 | TP 2 |  |  | TP 1 | TP 2 |  |  |
|  | median percentage  (range) | |  |  | median percentage  (range) | |  |  | median percentage  (range) | |  |  |
| **Tregs of CD4+ cells** | 0.82  (0.57-2.68) | 1.19  (0.99-3.11) | 45.1 | 0.297 | 1.26  (0.63-2.96) | 1.67  (0.67-4.73) | 32.5 | **0.0049** | 1.20  (0.28-3.84) | 1.40  (0.34-3.28) | 16.6 | 0.983 |
| **Tregs of total lymphocytes** | 0.34  (0.27-0.91) | 0.57  (0.34-1.66) | 67.6 | 0.219 | 0.32  (0.08-1.32) | 0.53  (0.11-1.49) | 65.6 | **0.0049** | 0.33  (0.02-0.79) | 0.32  (0.16-0.74) | -3.0 | 0.587 |

P-values result from comparing TP1 vs. TP2 values by Wilcoxon test.

*tp = time point
